# Analyzing the 3D chromatin organization coordinating with gene expression regulation in B-cell lymphoma

**DOI:** 10.1186/s12920-018-0437-8

**Published:** 2019-03-20

**Authors:** Luis Augusto Eijy Nagai, Sung-Joon Park, Kenta Nakai

**Affiliations:** 1 Department of Computational Biology and Medical Science, the University of Tokyo, 5-1-5 Kashiwanoha, Kashiwa-shi, Chiba 277-8562 Japan; 2 Human Genome Center, the Institute of Medical Science, the University of Tokyo, 4-6-1 Shirokanedai Minato-ku, Tokyo, 108-8639 Japan

**Keywords:** Chromatin organization, Transcriptome, Lymphoma, B cell, Hi-C

## Abstract

**Background:**

Eukaryotes compact chromosomes densely and non-randomly, forming three-dimensional structures. Alterations of the chromatin structures are often associated with diseases. In particular, aggressive cancer development from the disruption of the humoral immune system presents abnormal gene regulation which is accompanied by chromatin reorganizations. How the chromatin structures orchestrate the gene expression regulation is still poorly understood. Herein, we focus on chromatin dynamics in normal and abnormal B cell lymphocytes, and investigate its functional impact on the regulation of gene expression.

**Methods:**

We conducted an integrative analysis using publicly available multi-omics data that include Hi-C, RNA-seq and ChIP-seq experiments with normal B cells, lymphoma and ES cells. We processed and re-analyzed the data exhaustively and combined different scales of genome structures with transcriptomic and epigenetic features.

**Results:**

We found that the chromatin organizations are highly preserved among the cells. 5.2% of genes at the specific repressive compartment in normal pro-B cells were switched to the permissive compartment in lymphoma along with increased gene expression. The genes are involved in B-cell related biological processes. Remarkably, the boundaries of topologically associating domains were not enriched by CTCF motif, but significantly enriched with Prdm1 motif that is known to be the key factor of B-cell dysfunction in aggressive lymphoma.

**Conclusions:**

This study shows evidence of a complex relationship between chromatin reorganization and gene regulation. However, an unknown mechanism may exist to restrict the structural and functional changes of genomic regions and cognate genes in a specific manner. Our findings suggest the presence of an intricate crosstalk between the higher-order chromatin structure and cancer development.

**Electronic supplementary material:**

The online version of this article (10.1186/s12920-018-0437-8) contains supplementary material, which is available to authorized users.

## Background

To define three-dimensional (3D) chromatin structures in eukaryotic nuclei, Chromosome Conformation Capture (3C) sequencing technologies, such as the genome-wide 3C version (Hi-C), have emerged as a promising strategy and revealed that the 3D structures non-randomly compacted have functional roles for gene expression [1–5]. For example, in B cells (B lymphocytes), the nuclear lamina interacting directly and indirectly with the DNA and chromatin are disrupted during early lymphocyte development [6]. Another study [7] combining 3D fluorescence in situ and Hi-C analysis has shown that particular genome-wide structural transformations, such as the switching of chromatin compartments, are strongly linked with changes in transcription signatures in B cell development. In addition, the recent advancement in 3C technologies enables the identification of sub-compartment regions associated with B-cell fate determination [8].

B cells are central in the humoral immune system, and abnormal gene regulation in the cells is highly associated with cancer development [9]. Diffuse large B-cell lymphoma, one of the most common type of cancer in B cells, represents 30–40% of all non-Hodgkin lymphomas. Genetic translocations on the chromosome structure deregulate B Cell CLL/Lymphoma 6 (Bcl6) gene in germinal-center response in mice giving rise to different types of lymphoma [10]. Moreover, a recent study [11] using gene expression profiling revealed that PRDM1/BLIMP-1, a master regulator of plasma-cell differentiation, is inactivated in lymphoma where loss of genetic expression correlates with tumor cell proliferation.

Here, we sought to identify the chromatin dynamics involved in the gene regulation of B-cell lymphoma. We combined different scales of genome structures from Hi-C of published data [2, 7, 12] with gene expression profiles (RNA-seq) of mice. We observed that the higher-order chromatin organizations characterized as compartments and topologically associating domains (TADs) are highly conserved among cells. Moreover, these compartments switch from repressive to permissive in pro-B cells and lymphoma and exhibit increased gene expression levels in comparison with ES cells. However, the switch of the repressive compartment in B cell to the permissive in lymphoma (~ 5.2% of the genes) have portrayed overall fluctuation of gene expression level regardless of the compartment dynamics. Interestingly, TAD boundaries are enriched with Prdm1 motif, suggesting a possibility of coordination between the higher-order of chromatin structures and cancer development.

## Methods

### Data preparation

RNA-seq datasets were downloaded from Gene Expression Omnibus (GEO): (i) GSM2698041 and GSM2698042 for mouse embryonic stem cells (ES cells), cell type 129S4/SvJae, sex not informed, from total RNAs; (ii) GSM1897405, GSM1897406, and GSM1897407 for normal B cells in mice, cell type C57BL/6 pro-B cell, pool of male and female individuals, from total RNAs; (iii) GSM2072416 and GSM2072417 for mouse B-cell lymphoma from strain B10.H-2aH-4 bp/Wts CH12.LX immortalized cell line, female, from total RNAs. Hi-C data were downloaded from GEO: (i) GSM862720 and GSM862721 for mouse ES cells, cell type 129S4/SvJae, HindIII restriction enzyme, male, from genomic DNAs; (ii) GSM987818 for mouse pro-B cells, sex not informed, C57bl/6, HindIII restriction enzyme, from genomic DNAs; (iii) GSE63525 for mouse B-cell lymphoma, CH12-LX immortalized cell line, MboI restriction enzyme, sex not informed, from genomic DNAs. The detailed information on quality controls and mapping ratios can be found in Additional file 1: Tables S1-S4

### RNA-seq data analysis

Firstly, sequencing reads were trimmed by Trimmomatic 0.36 (with the parameters: ILLUMINACLIP:TruSeq3-SE.fa:2:30:10 LEADING:3 TRAILING:3 SLIDINGWINDOW:4:15 MINLEN:36) [13]. The processed reads were aligned to mm10 cDNA and counted by Salmon [14] (with the parameters salmon quant -i < mm10 cdna> − l A). Gene abundances were obtained from transcripts by R/Bioconductor package tximport [15]. The detailed information is in Additional file 1: Tables S1 and S2.

### Hi-C data analysis

#### Hi-C matrix

Paired-end Hi-C reads were trimmed by Trimmomatic (ILLUMINACLIP:TruSeq3-PE.fa:2:30:10 LEADING:3 TRAILING:3 SLIDINGWINDOW:4:15 MINLEN:25) and were mapped separately to mm10 by BWA-mem [16]. In order to consider chimeric reads, we performed BWA-mem with a gap extension penalty and clipping at 5′ and 3′ ends (−A 1 -B 4 -E 50 -L 0 -T 25 -t 10), which allows the aligner to divide chimeric reads and to map the two parts of the read separately. HiCExplorer 2.0 [17] built Hi-C matrices with read counts over the bins of unequal size considering restriction sites; HindIII (AAGCTT) for pro-B cell and ES cell, and MboI (GATC) for B cell lymphoma. Briefly, the values of rows and columns in a Hi-C matrix stand frequencies that any two bins were connected by any pairs of processed read. This process discards non-uniquely mapped reads, lower mapping score reads, duplicated, re-ligation and dangling ends. To avoid amplification biases, low count bins and higher outliers were filtered out by setting a threshold on bimodal distribution. Hi-C replicates of each sample were merged as recommended by the HiCExplorer manual. To avoid the sex dependent bias, we removed chromosome Y from Hi-C merged matrices. Then, iterative correction was performed as described in Imakaev et al. [18]. The detailed information can be found from Additional file 1: Tables S3 and S4.

#### Compartment identification and TAD calling

HOMER [19] performed the principal component analysis (PCA) on normalized interaction matrices and integrated H3K36me3 peaks to assign positive values to A compartment and negative values to B compartment. We downloaded ChIP-seq BED files from ENCODE [20]: ENCSR000CGR for ES cells, ENCSR000CFY for B cells, and ENCSR000CFL for B-cell lymphoma. To identify TADs, we ran the program “hicFindTADs” of HiCExplorer; it first transforms the Hi-C contact matrix into a z-score matrix considering all contacts at the same genomic distance. Then, separation scores are computed for different values of window, and low scores are indicative of TAD boundaries. To compare submatrix values, Wilcoxon rank-sum test was applied, and the *p*-values were corrected by Bonferroni method. The boundaries with adjusted *p* < 0.01 were reported.

### Bioinformatics analysis

#### Classification of mouse genes per compartment

To avoid redundancy in counting and assigning to both compartments, we considered only transcription start site (TSS) positions of genes. By using the program “intersect” in bedtools [21] with the parameter “-wo -F 1.0”, we prepared genes whose TSSs were overlapped with either A or B compartments.

#### Gene ontology enrichment analysis

We conducted gene ontology (GO) enrichment analysis using DAVID [22]. We first compartmentalized the genome described above and prepared gene sets that were located in different compartments in a pair of cells. We analyzed GO biological process terms for each gene set. We used 0.05, 0.01 as the thresholds for *P*-values and EASE score, respectively. We listed additional GO terms in supplementary files using Bonferroni correction with the threshold 0.05 (Additional file 1: Table S6-S9).

#### Calculating normalized scores

We normalized the scores for compartments per samples; for each chromosome in a sample, A or B compartment count is divided by the total number of compartments in the respective chromosome, and is divided by the respective chromosome size. We also normalized the scores for TADs in the same manner.

#### Motif enrichment analysis

We used the program HOMER [19] to perform the motif enrichment analysis on TAD-boundary sequences. We used 20 Kb (kilo base) upstream and downstream DNA sequences of a TAD boundary. Significant sequence motifs (*p* < 0.01) found by HOMER were reported.

## Results

### Highly conserved folding patterns on chromatin compartment domains

Eukaryotic genomes are composed of sets of loci that are more likely to interact with one another than expected by random conformation of a chromosome. These sets show a plaid pattern that classify each genomic locus into either A or B compartments [23]. Interaction maps from Hi-C data can provide information in multiple levels of genome organization hierarchy [24]. The first level to examine chromatin interactions is the compartment domain. To examine the 3D chromatin folding dynamics in B cell and lymphoma, we prepared public Hi-C data of pro-B cells [7], B-cell lymphoma [12], and ES cells [2]. Then, we performed PCA analysis with higher resolution (100 Kb) on the mouse genome.

**Fig. 1:**
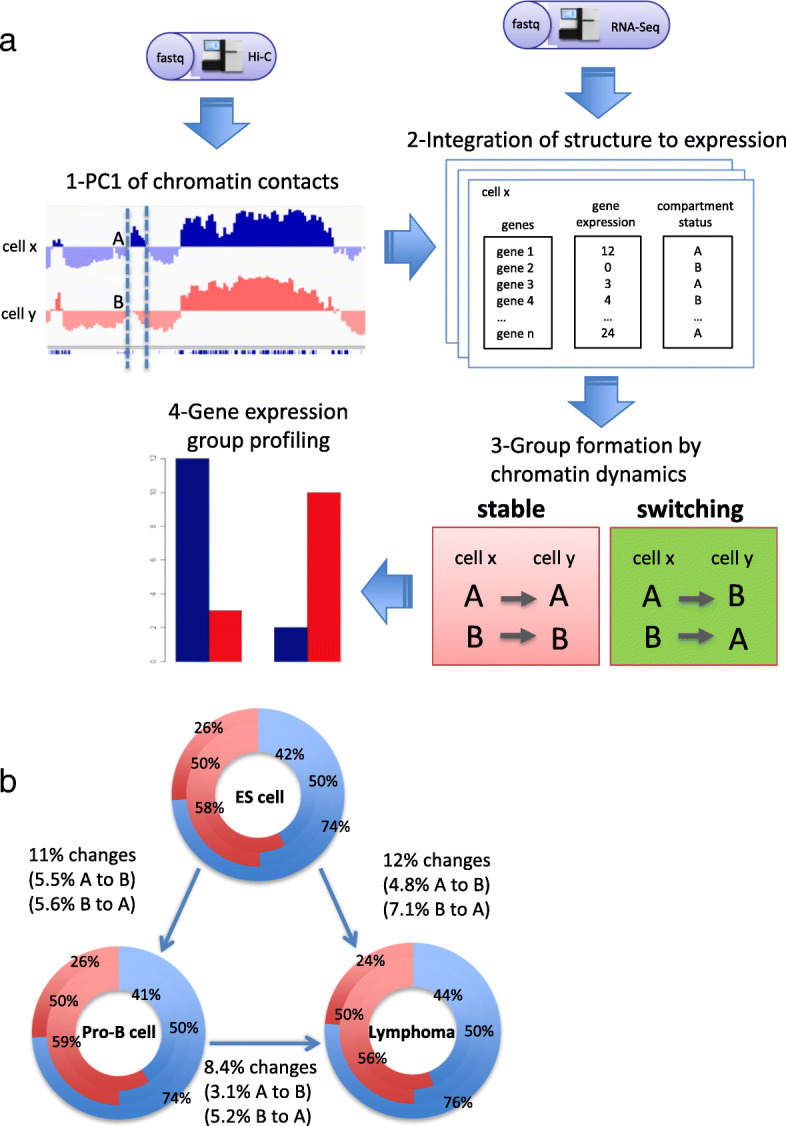
**a** Representation of our integrative analysis. First, we obtained the first principal component (PC1) values from Hi-C chromatin maps and assigned each gene to active (A compartment) or inactive (B compartment) in each cell. Next, we included gene expression levels to the matrix profiles. Compartment classification of each gene in two cell types categorized four compartment switch groups (i.e., stable A, A to B, B to A, and stable B). Stable regions have the same compartments in two different cells. Finally, we obtained the overall gene expression levels on the groups. **b** Chromatin organization characterization at compartment level in ES cells, pro-B cells, and Lymphoma. Each graph represents the compartment landscape; blue is for A and red is for B. The inner layer represents the percentage of genomic sequences within A or B compartments; middle layer represents the ratio of A and B compartments; outer layer represents the percentage of genes contained in each compartment

Overall, our analysis classified the genome into ~ 1.48 Gb of B compartment and ~ 1.1 Gb of A compartment in pro-B cells. The A and B compartments contained 14,600 and 4900 genes, respectively. To compare the folding patterns among the cells, we compared the A and B coordinates as described previously in [25] (Fig. 1a). We found that 89 and 88% of the genomic coordinates, in pro-B cell and lymphoma respectively, remained in the same compartment or stable status as compared to the ES cell (both in A or both in B) [26] (Fig. 1b). Furthermore, we found a higher similarity (91.6%) between pro-B cell and lymphoma compartment coordinates. These results are consistent with the observation in a study that shows 90.7% of the compartments are conserved in pre-pro-B and pro-B mice cells [25]. A previous study with human cells also found a similarity degree of 64% among ES cells and four derived lineage compartments [26]. While in another study conducted across 21 human cells and tissues, researchers observed 40.4% of conservation in the compartments [27]. These results suggest that our analysis achieves satisfactory chromatin organization structures by finding very similar chromatin compartment domains between pro-B cell and lymphoma using heterogeneous data resources.

### Extensive reorganization of the mouse genomic compartments and the impact on gene expression levels

It has been known that compartment reorganizations are associated with the disruption of normal gene expression program leading to breast cancer [28]. In order to investigate whether this phenomenon is also observed in mouse B cell-derived lymphoma, we identified chromatin compartments, at 100 Kb resolution, from normalized chromatin interaction matrices and obtained gene expression values from RNA-seq data. We examined switch regions between compartments across the cells (Fig. 1a-b). Within the regions, we found genes related to B-cell development functions, B-cell lymphoma and early embryonic stages. For example, Ebf1 that is an important regulator for B cell fate [25] and IgHa that has a potential role in lymphoma development [29] changed the compartment states from inactive B compartment in ES cells to active A compartment both in pro-B cell and lymphoma with increased gene expression changes. Bcas1 also shows evidence of coordination in early activation of restricted transcription in ES cells [30]. In contrast, Myc that is associated with translocations and gene amplifications in B-cell lymphoma [31] was changed from inactive compartment in ES cells to active compartment in lymphoma without changing the expression level. Hdac9, which is a chromatin-modifying enzyme functioning in early stages of B-cell development [32], showed B-to-A compartment activation with the positive correlation of expression level in pro-B cells but the negative correlation of expression level in lymphoma.

**Fig. 2:**
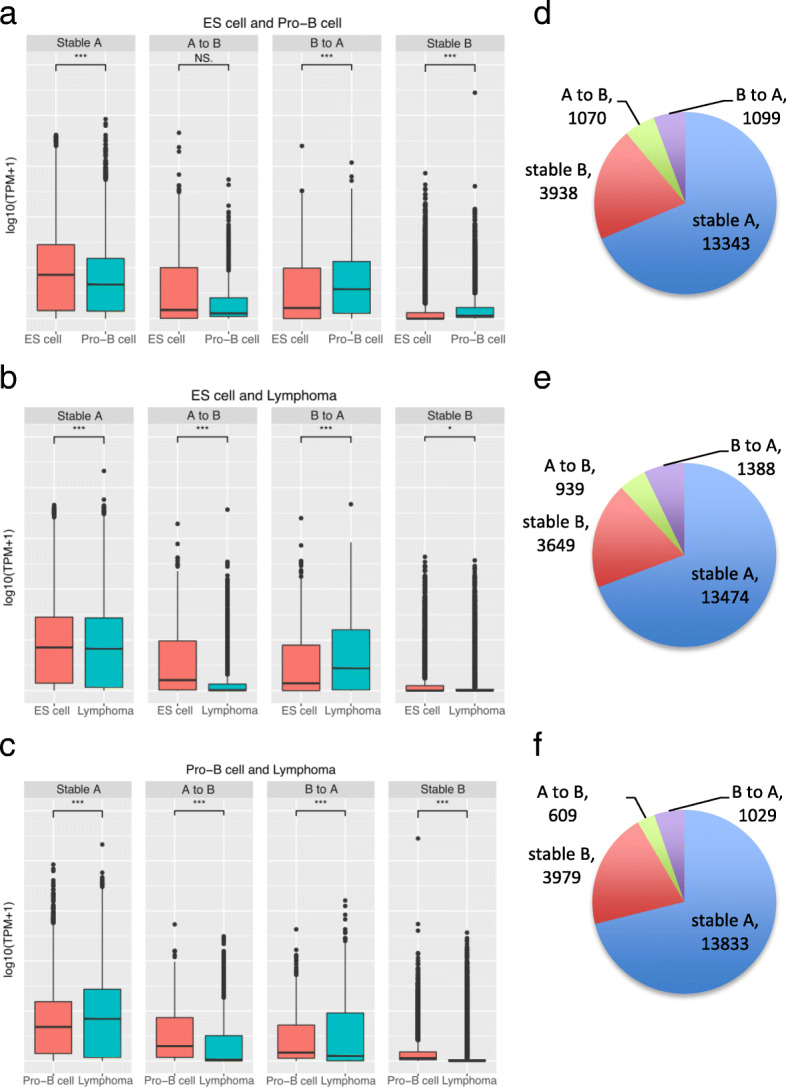
Profile of gene expression in compartment reorganization comparing ES cells, pro-B cells and lymphoma. Values represent the expression level of genes identified in the respective regions (log10 of TPM values + 1). The panels (**a**-**c**) show the tendency of genes having higher RNA abundance when located at active A compartment rather than inactive B compartment. Although B compartment is extensively reported as inactive compartment, it still contains genes with higher levels of RNA molecules. **a** ES cells and pro-B cells comparison. **b** ES cells and lymphoma comparison. **c** Pro-B cells and lymphoma comparison. The plots (**d**-**f**) represent the ratio of each comparison with the number of genes in each group

**Fig. 3:**
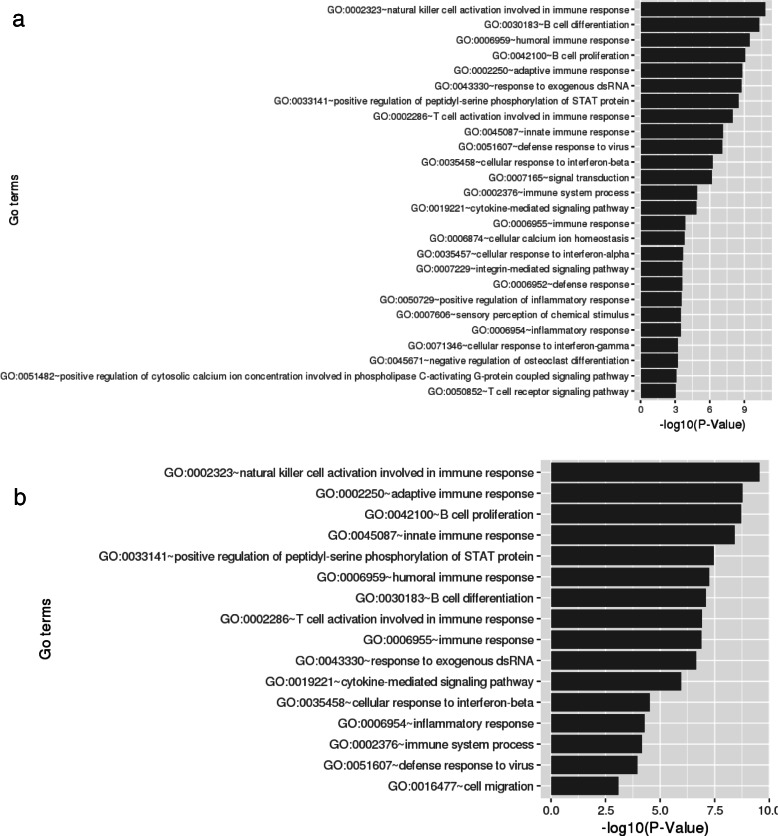
Enriched GO biological process terms in set of genes from compartment reorganization. **a** Results from set of all genes in switching compartment region from B (ES cells) to A (pro-B cells). **b** Results from set of all genes in switching compartment region from B (ES cells) to A (B-cell lymphoma)

**Fig. 4:**
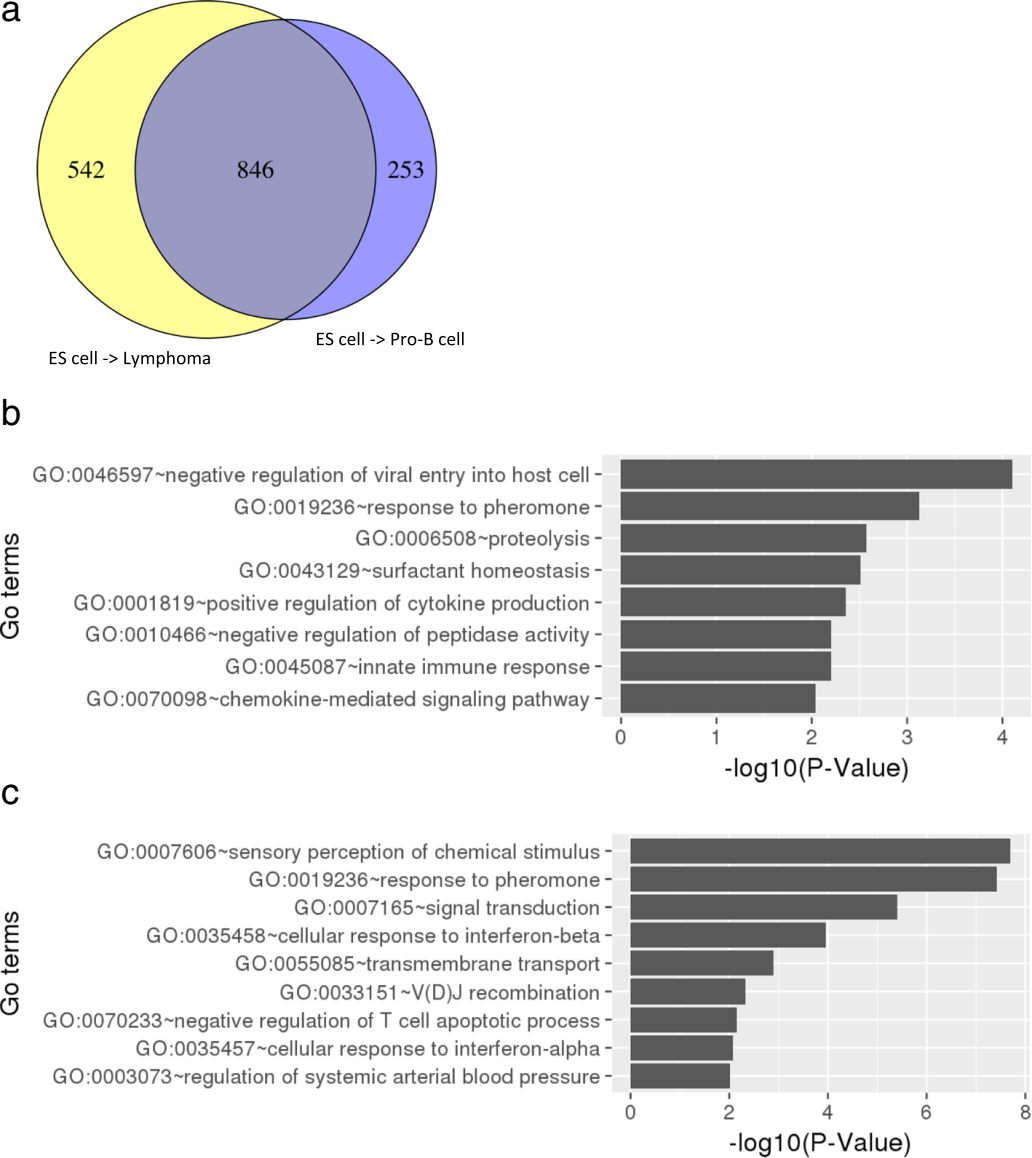
**a** Venn diagram showing the distribution of genes in B-to-A compartment changes. **b** GO term enrichment in the genes unique in switching compartment region from B (ES cells) to A (Pro-B cells). **c** GO term enrichment in the genes unique in switching compartment region from B (ES cells) to A (B-cell lymphoma)

To investigate the influence of chromatin compartmentalization frequency in chromosomes, we calculated a normalization score by dividing the sum of compartments in each chromosome by its chromosome size. As expected, the distribution of compartments throughout the genome was much more similar between pro-B cells and lymphoma than between those cells and ES cells (Additional file 2: Figure S1). The genes located in B compartment in ES cells switching to A compartment both in pro-B cells and lymphoma tend to show increased gene expression levels, whereas the genes positioned in A-to-B compartment change show the opposite tendency (Fig. 2a-c). The tendency was not observed from B-to-A change in pro-B cells to lymphoma. This suggests that the overall tendency of gene expression in compartment changes is not absolute as only a part of genes are affected by the compartment changes, and the other part may receive influence of other factors not covered by our approach [26, 28]. Since the limited number of genes were contained in the B-to-A compartment activations (Fig. 2d-f), we further compared the gene expression levels of switching-genes with those of random genes located in stable regions (Additional file 2: Figure S2). To analyze functional enrichments, we selected all the genes that were involved in B-to-A compartment change. The genes in pro-B cells and lymphoma were enriched for similar GO terms related to B cell function, such as natural killer cell activation involved in immune response, humoral immune response, B cell proliferation, and immune response process (Fig. 3 and Additional file 1: Table S6 and S7). After discarding 846 genes common in the cells (Fig. 4a), we found that genes in pro-B cells were enriched with immune response terms including negative regulation of viral entry into host cell and proteolysis (Fig. 4b and Additional file 1: Table S8). Meanwhile, genes in lymphoma were enriched with sensory perception of chemical stimulus, V(D)J recombination, and negative regulation of T cell apoptotic process (Fig. 4c and Additional file 1: Table S9).

Our results support the evidence of intricate relationship between differential chromosomal structure changes and gene expression. Interestingly, the genes that switched from B compartment in ES cells to A compartment in both pro-B cells and lymphoma were identified to be strongly related to B-cell biological processes, although not completely represented by our gene sets. Thus, by using chromatin organization coordinates, we can provide meaningful insights to clarify the functions of specific genes. In addition, in general, the compartment changes correspond to changes in gene expression levels, indicating that A and B compartments might be involved in the orchestration of gene regulation.

### Influence of topologically associating domains on compartment reorganization

We next examined the sub-compartment structures known as TADs [2], organizing dense and contiguous self-interacting regions. Although TADs tend to be conserved across different types of cells, chromatin interactions vary from cells to cells [26]. Here we raise the question about the possibility that TADs contribute to the gene expression programs in pro-B cells and lymphoma.

**Fig. 5:**
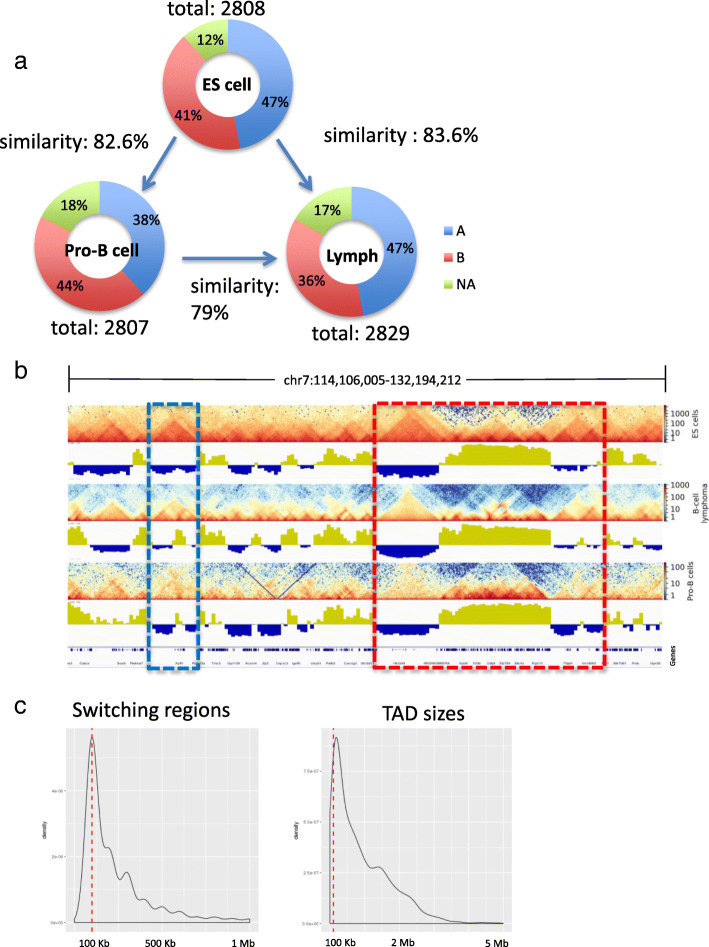
**a** Chromatin organization at TAD level in ES cells, Pro-B cells, and Lymphoma. TADs were classified per compartment, in which, A compartments are in blue, B compartments in red, and TADs between two compartments are in green. The degree of similarity is shown by the arrow between two cell types (**b**) An example of the 3D chromatin structures including unique TADs (blue square) and conserved regions (red square) among cell types. The heatmaps represent TADs per cell type; compartments are represented by the PC1 values, which the positive values indicate the A compartment, and negative values represent the B compartment. **c** Distribution of the overall ranges of switching compartment regions and TAD sizes

At 40 Kb resolution, our TAD calling classified the genome structures into similar numbers; 2829 in lymphoma, 2807 TADs in pro-B cells, and 2808 in ES cells. Interestingly, the majority of TADs identified in a cell was conserved in another cell; by applying the approach described previously [33], we identified > 70% overlapped TADs in a pair of samples, resulting in 2348 (83.6% of total TADs) in lymphoma and ES cells, 2319 (82.6%) in pro-B cell and ES cells, 2235 (79%) in lymphoma and pro-B cell (Fig. 5a). These observations suggest that chromosomes retain their physical conformation within the nucleus. In all samples, TADs were largely identified from chromosome 7. Lymphoma formed a notably larger number though in smaller sizes of TADs in chromosome 14, and smaller number of TADs in chromosome X (Additional file 2: Figure S3). A previous study has attributed the smaller sizes of TADs in prostate cancer to the establishment of shorter distances within TAD boundaries [34]. This observation suggests that the similar mechanism in lymphoma may access different genomic loci by different interactions from distinct TAD formations. Herein, we have identified 301 unique TADs in pro-B cell, 191 in ES cell, and 198 in lymphoma (Fig. 5b), in which the ratio of unique TADs in pro-B cells is similar to those found in a previous study; 65 unique out of 787 TADs identified in pro-B cell [25].

In order to investigate whether TADs present any relationship to compartment changes, we have compared TAD coordinates and switching regions. Surprisingly, a great number of TADs were included in the 100-Kb-switching regions (Fig. 5b and c). For example, 662 (23.6%) pro-B cell TADs were identified within switching regions between ES cell and pro-B cell, 486 (17.3%) lymphoma TADs were identified between ES cell and lymphoma, and 306 (10.8%) lymphoma TADs were found within switching regions between pro-B cell and lymphoma. This suggests that 10–20% of the overall TADs, ranging at about ~ 100 Kb, are located in regions of dynamic change of interactions.

### TAD boundaries suggest gene regulation function in cancer

Recent studies have revealed that TADs associate with CTCF and cohesin [35] by forming relative conserved structures across cell types [36] to bring enhancers and specific genes closer [12]. Also, it has been observed that the disruption of TAD boundaries promotes gene expression leading to a physical malformation in mice [37], suggesting the importance of CTCF in TAD boundaries. On the other hand, only 15% of CTCF motifs are located at TAD boundaries in mammals and 85% reside inside TADs [38]. This scattered disposition points out that whereas CTCF can afford flexible adjustment to the chromatin conformation, the 3D chromatin organization is more likely to be influenced by a fine orchestration of cell-specific regulatory program. We then asked whether TAD boundaries of normal and cancer cells would exhibit CTCF enrichment [17, 39, 40], and whether genes located at the boundaries would exhibit any variation in gene expression levels when compared to those located at within-TAD regions.

**Fig. 6:**
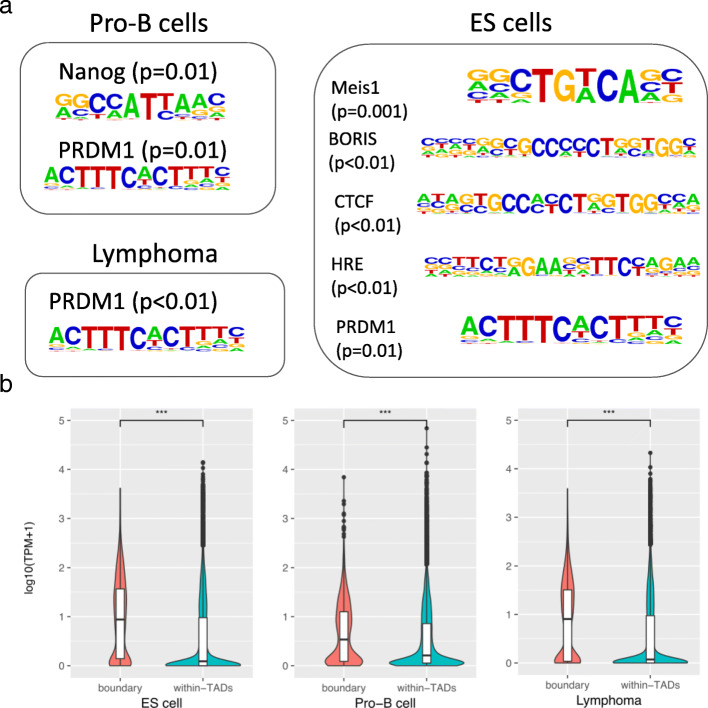
(**a**) Motifs identified at the TAD boundaries. (**b**) Comparison of gene expression values based on the chromatin structure disposition of TADs. Values are normalized gene expression values (TPM) represented in log10

We observed CTCF and BORIS motifs from the TAD boundaries in ES cells (*p* < 0.01). We also found HRE (*p* < 0.01) and Meis Homeobox 1 enrichments (*p* = 0.001), which are known to play important roles in normal mouse development [11]. The TAD boundaries in pro-B cell were enriched with Nanog and PRDM1 motifs (*p* = 0.01). Interestingly, the coding gene of Prdm1 that is associated with various cancer developments [11, 37–39, 41–43] exhibited the high expression level only in lymphoma (Additional file 2: Figure S4), even though all samples included PRDM1 in A compartment (Additional file 1: Table S5) enriched in TAD boundaries (Fig. 6a). We could not profile the CTCF motif enrichment in neither lymphoma nor pro-B cells.

Next, we categorized genes based on their proximity to TAD boundaries. Remarkably, genes located around TAD boundaries (<40Kb) showed significantly higher gene expression levels (*p* < 0.0001) in all samples (Fig. 6b). This has also been observed in a high-resolution experiment in fruit flies [17]. Overall, although TADs are highly conserved between cell types and are often delimited by CTCF motifs, our results show the relationship of TAD boundaries with cancer-related transcription factors rather than with CTCF.

## Discussion

Recent studies have revealed that the eukaryotic genome is divided into chromatin compartments and boundary-limited functional units known as TADs. This genomic architecture inside the cell nucleus exhibits conserved folding patterns across species and cell types. The current model proposes that CTCF may guide the chromatin folding to form intradomains inside compartments [44]. The chromatin-folding dynamics coordinates with transcriptional changes, but the underlying mechanisms are still poorly understood. Here, we used publicly available libraries of Hi-C paired-end sequences from three different resources to compare general chromatin structures. To quantify the chromatin structures, we designed a method coupled with the approach described by Hi-C developers [23], which is non-sensitive to compartment identification at higher resolution analyses [45]. Overall, we identified among samples similar numbers of compartments and TADs per chromosome which suggests a strong identifiability of chromatin folding. In addition, we observed several 100–200 kilobases compartments, which supports recent studies that report kilo-base-sized compartments [46, 47].

Our analysis revealed the genomic regions that switch compartment status in the context of cells: 8.4% between pro-B cell and lymphoma, 11% between pro-B cells and ES cell, and 12% between lymphoma and ES cell. These regions included specific genes: 1091 genes in pro-B cells, 1378 genes in lymphoma. These genes were located in compartments that switch from inactive/silenced to active/transcribed status. The functional annotation analysis revealed that the genes possess B-cell specific functions. Most importantly, not all genes identified in the chromatin reorganization regions had the gene expression levels coordinating with compartment dynamics. For example, Bcl6 and Bcl11a, which are marker genes for lymphoma, showed higher gene expression levels in ES cells, even though located in B compartment in ES cells. This suggests that more research is needed to understand the exact mechanism of the process.

Collectively, our results show that the majority of TADs among pro-B cells, lymphoma and ES cells are highly conserved yet exhibiting some identity as previously reported [48], whereas specific genomic regions are involved in the structural reorganization. We observed CTCF motifs enriched only at TAD boundaries in ES cells, which is consistent with a previous report [44]. However, we demonstrated that the DNA sequences at TAD boundaries are not always related to CTCF. The enrichment of PRMD1 motif found in this study encourages further effort to investigate the association of cancer-related motifs with TAD structures.

## Conclusions

Our results show that the majority of TADs among pro-B cells, lymphoma and ES cells are highly conserved, whereas specific genomic regions are involved in the compartment change. In particular, the switching compartment regions are followed by a subtle gene expression increased between pro-B cell and lymphoma when compared to ES cell. We concluded that an unknown mechanism may exist to restrict the structural and functional changes of genomic regions and cognate genes in a specific manner.

## Additional files

Additional file 1: This file includes: detailed information from data used, including statistical information of reads and mapping process in RNA-seq and Hi-C analysis (**Table S1-S4**), gene expression values from heatmap in Additional file 2: Figure S4 (**Table S5**), gene ontology analysis detailed results (**Table S6-S9**). (XLSX 85 kb)

Additional file 2: This file includes: a distribution of normalized compartment scores per chromosome (**Figure S1**), gene expression profile of switching regions including random genes from stable regions (**Figure S2**), distribution of normalized topologically associating domain scores per chromosome (**Figure S3**), and heatmap of set of genes known to be involved in B-cell fate and B-cell lymphoma (**Figure S4**). (PPTX 11246 kb)

## Abbreviations

3C: Chromosome conformation capture
3D: Three-dimensional
CTCF: CCCTC-binding factor
ES cell: Embryonic stem cell
GO: Gene ontology
Hi-C: An extension of 3C
PCA: Principal component analysis
TAD: Topologically associating domain
TPM: Transcripts per million
TSS: Transcription start site

## References

[1] Jin F (2013). A high-resolution map of the three-dimensional chromatin interactome in human cells. Nature.

[2] Dixon JR (2012). Topological domains in mammalian genomes identified by analysis of chromatin interactions. Nature.

[3] Yue F (2014). A comparative encyclopedia of DNA elements in the mouse genome. Nature.

[4] Rudan MV (2015). Comparative hi-C reveals that CTCF underlies evolution of chromosomal domain architecture. CellReports.

[5] Jung YH (2017). Chromatin states in mouse sperm correlate with embryonic and adult regulatory landscapes. CellReports.

[6] Kosak ST, et al. Subnuclear compartmentalization of immunoglobulin loci during lymphocyte development. Science. 2002;296(5565):158–62. 10.1126/science.1068768 11935030

[7] Lin YC (2012). Global changes in the nuclear positioning of genes and intra-and interdomain genomic interactions that orchestrate B cell fate. Nat Immunol.

[8] Bonev B, Mendelson Cohen N, Szabo Q, Fritsch L, Papadopoulos GL, Lubling Y, Xu X, Lv X, Hugnot JP, Tanay A, Cavalli G. Multiscale 3D genome rewiring during mouse neural development. Cell. 2017;171(3):557–72.e24. 10.1016/j.cell.2017.09.043 PMC5651218 29053968

[9] Klein U, et al. Gene expression profiling of B cell chronic lymphocytic leukemia reveals a homogeneous phenotype related to memory B cells. J Exp Med. 2001;121400(11):1625–38. 10.1084/jem.194.11.1625 PMC2193527 11733577

[10] Lenz G, Staudt LM (2010). Aggressive lymphomas. N Engl J Med.

[11] Xia Y (2017). Loss of PRDM1/BLIMP-1 function contributes to poor prognosis of activated B-cell-like diffuse large B-cell lymphoma. Leukemia.

[12] Rao SS (2014). A 3D map of the human genome at kilobase resolution reveals principles of chromatin looping. Cell.

[13] Bolger AM, Lohse M, Usadel B. Genome analysis Trimmomatic : a flexible trimmer for Illumina sequence data. Bioinformatics. 2014;30(15):2114–20. 10.1093/bioinformatics/btu170 PMC4103590 24695404

[14] Patro R, Duggal G, Love MI, Irizarry RA, Kingsford C (2017). Salmon provides fast and bias-aware quantification of transcript expression. Nat Publ Gr.

[15] Soneson C, Love MI and Robinson MD. Differential analyses for RNA-seq: transcript-level estimates improve gene-level inferences. F1000Research. 2015;4:1521. 10.12688/f1000research.7563.1 PMC4712774 26925227

[16] Li H, Durbin R. Fast and accurate short read alignment with Burrows – Wheeler transform. Bioinformatics. 2009;25(14):1754–60. 10.1093/bioinformatics/btp324 PMC2705234 19451168

[17] Ramírez F (2018). High-resolution TADs reveal DNA sequences underlying genome organization in flies. Nat Commun.

[18] Imakaev M (2012). Iterative correction of Hi-C data reveals hallmarks of chromosome organization. Nat Methods.

[19] Heinz S (2010). Simple combinations of lineage-determining transcription factors prime cis-regulatory elements required for macrophage and B cell identities. Mol Cell.

[20] Dunham I (2012). An integrated encyclopedia of DNA elements in the human genome. Nature.

[21] Quinlan AR, Hall IM. BEDTools: a flexible suite of utilities for comparing genomic features. Bioinformatics. 2010;26(6):841–2. 10.1093/bioinformatics/btq033 PMC2832824 20110278

[22] Huang DW, Sherman BT, Lempicki RA (2009). Systematic and integrative analysis of large gene lists using DAVID bioinformatics resources. Nat Protoc.

[23] Lieberman-Aiden E, et al. Comprehensive mapping of long-range interactions reveals folding principles of the human genome. Science. 2009;326(5950):289–93. 10.1126/science.1181369 PMC2858594 19815776

[24] Gorkin DU, Leung D, Ren B (2014). The 3D genome in transcriptional regulation and pluripotency. Cell Stem Cell.

[25] Boya R, Yadavalli AD, Nikhat S, Kurukuti S, Palakodeti D, Pongubala JMR. Developmentally regulated higher-order chromatin interactions orchestrate B cell fate commitment. Nucleic Acids Res. 2017;45(19):11070–11087. 10.1093/nar/gkx722 PMC5737614 28977418

[26] Dixon JR (2015). Chromatin architecture reorganization during stem cell differentiation. Nature.

[27] Schmitt AD (2016). A compendium of chromatin contact maps reveals spatially active regions in the human genome. Cell Rep.

[28] Barutcu AR (2015). Chromatin interaction analysis reveals changes in small chromosome and telomere clustering between epithelial and breast cancer cells. Genome Biol.

[29] Cleary ML, Chao J, Warnke R, Sklar J (1984). Immunoglobulin gene rearrangement as a diagnostic criterion of B-cell lymphoma. Proc Natl Acad Sci U S A.

[30] Fard MK (2017). BCAS1 expression defines a population of early myelinating oligodendrocytes in multiple sclerosis lesions. Sci Transl Med.

[31] Nguyen L, Papenhausen P, Shao H. The role of c-MYC in B-cell lymphomas: diagnostic and molecular aspects. Genes (Basel). 2017;8(4):116. 10.3390/genes8040116 PMC5406863 28379189

[32] Gil VS (2016). Deregulated expression of HDAC9 in B cells promotes development of lymphoproliferative disease and lymphoma in mice. Dis Model Mech.

[33] Wu P (2017). 3D genome of multiple myeloma reveals spatial genome disorganization associated with copy number variations. Nat Commun.

[34] Taberlay PC (2016). Three-dimensional disorganisation of the cancer genome occurs coincident with long range genetic and epigenetic alterations. Genome Res.

[35] Zuin J (2014). Cohesin and CTCF differentially affect chromatin architecture and gene expression in human cells. Proc Natl Acad Sci.

[36] Schwarzer W (2017). Two independent modes of chromatin organization revealed by cohesin removal. Nature.

[37] Lupiáñez DG (2015). Disruptions of topological chromatin domains cause pathogenic rewiring of gene-enhancer interactions. Cell.

[38] Ruiz-Velasco M, Zaugg JB (2017). Structure meets function: how chromatin organisation conveys functionality. Curr Opin Syst Biol.

[39] Kaiser VB, Semple CA (2018). Chromatin loop anchors are associated with genome instability in cancer and recombination hotspots in the germline. Genome Biol.

[40] Eser U, Chandler Brown D, Ay F, Straight AF, Duan Z, Noble WS, Skotheim JM. Form and function of topologically associating genomic domains in budding yeast. Proc Natl Acad Sci USA. 2017;114:E3061–E3070. 10.1073/pnas.1612256114 PMC5393236 28348222

[41] Zhang Z (2017). Hypermethylation of PRDM1/Blimp-1 promoter in extranodal NK/T-cell lymphoma, nasal type: an evidence of predominant role in its downregulation. Hematol Oncol.

[42] Zhu Z, Wang H, Wei Y, Meng F, Liu Z, Zhang Z. Downregulation of PRDM1 promotes cellular invasion and lung cancer metastasis. Tumor Biol. 2017;39(4). 10.1177/1010428317695929 28378641

[43] S. Kang et al., Adequate concentration of B cell leukemia/lymphoma 3 (Bcl3) is required for pluripotency and self-renewal of mouse embryonic stem cells via downregulation of Nanog transcription. BMB Rep. 2018;51(2):92–97. 10.5483/BMBRep.2018.51.2.219 PMC5836563 29335071

[44] Nora EP (2017). Targeted degradation of CTCF decouples local insulation of chromosome domains from genomic compartmentalization. Cell.

[45] Fortin J-P, Hansen KD (2015). Reconstructing A/B compartments as revealed by Hi-C using long-range correlations in epigenetic data. Genome Biol.

[46] Wang Q, Sun Q, Czajkowsky DM, Shao Z (2018). Sub-kb Hi-C in D. melanogaster reveals conserved characteristics of TADs between insect and mammalian cells. Nat Commun.

[47] Rowley MJ (2017). Evolutionarily conserved principles predict 3D chromatin organization. Mol Cell.

[48] Forcato M, Nicoletti C, Pal K, Livi CM, Ferrari F, Bicciato S (2017). Comparison of computational methods for hi-C data analysis. Nat Methods.

