# Supplementary material for: Analyzing the 3D chromatin organization coordinating with gene expression regulation in B-cell lymphoma
Source: BMC Med Genomics. 2019 Mar 20;11(Suppl 7):127. doi: 10.1186/s12920-018-0437-8 (PMC7402584; doi:10.1186/s12920-018-0437-8)
Supplement: Supplementary file 2 — This file includes: a distribution of normalized compartment scores per chromosome (Figure S1), gene expression profile of switching regions including random genes from stable regions (Figure S2), distribution of normalized topologically associating domain scores per chromosome (Figure S3), and heatmap of set of genes known to be involved in B-cell fate and B-cell lymphoma (Figure S4). (PPTX 11246 kb) [file 12920_2018_437_MOESM2_ESM.pptx]

## Slide 1
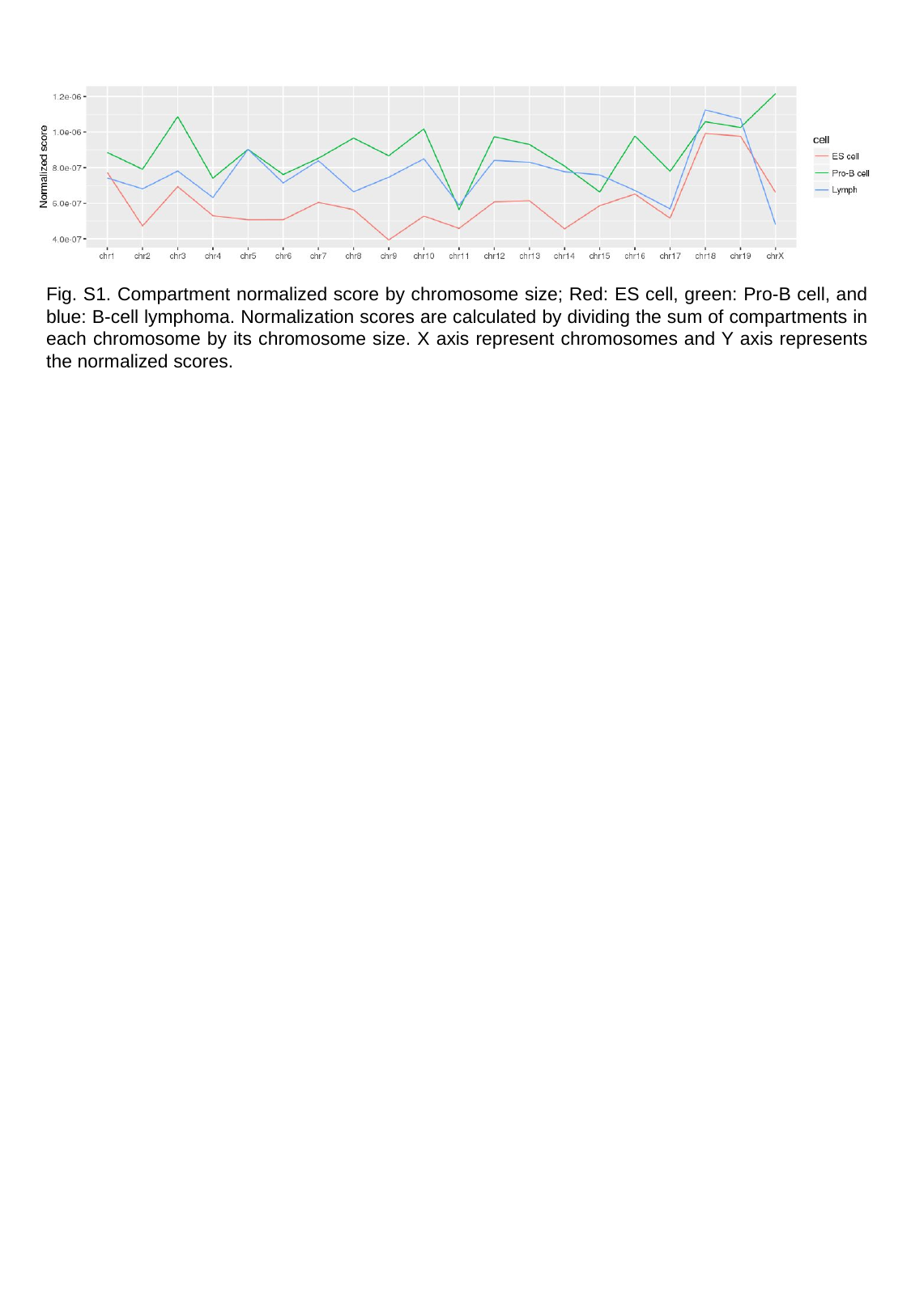

Fig. S1. Compartment normalized score by chromosome size; Red: ES cell, green: Pro-B cell, and blue: B-cell lymphoma. Normalization scores are calculated by dividing the sum of compartments in each chromosome by its chromosome size. X axis represent chromosomes and Y axis represents the normalized scores.

## Slide 2
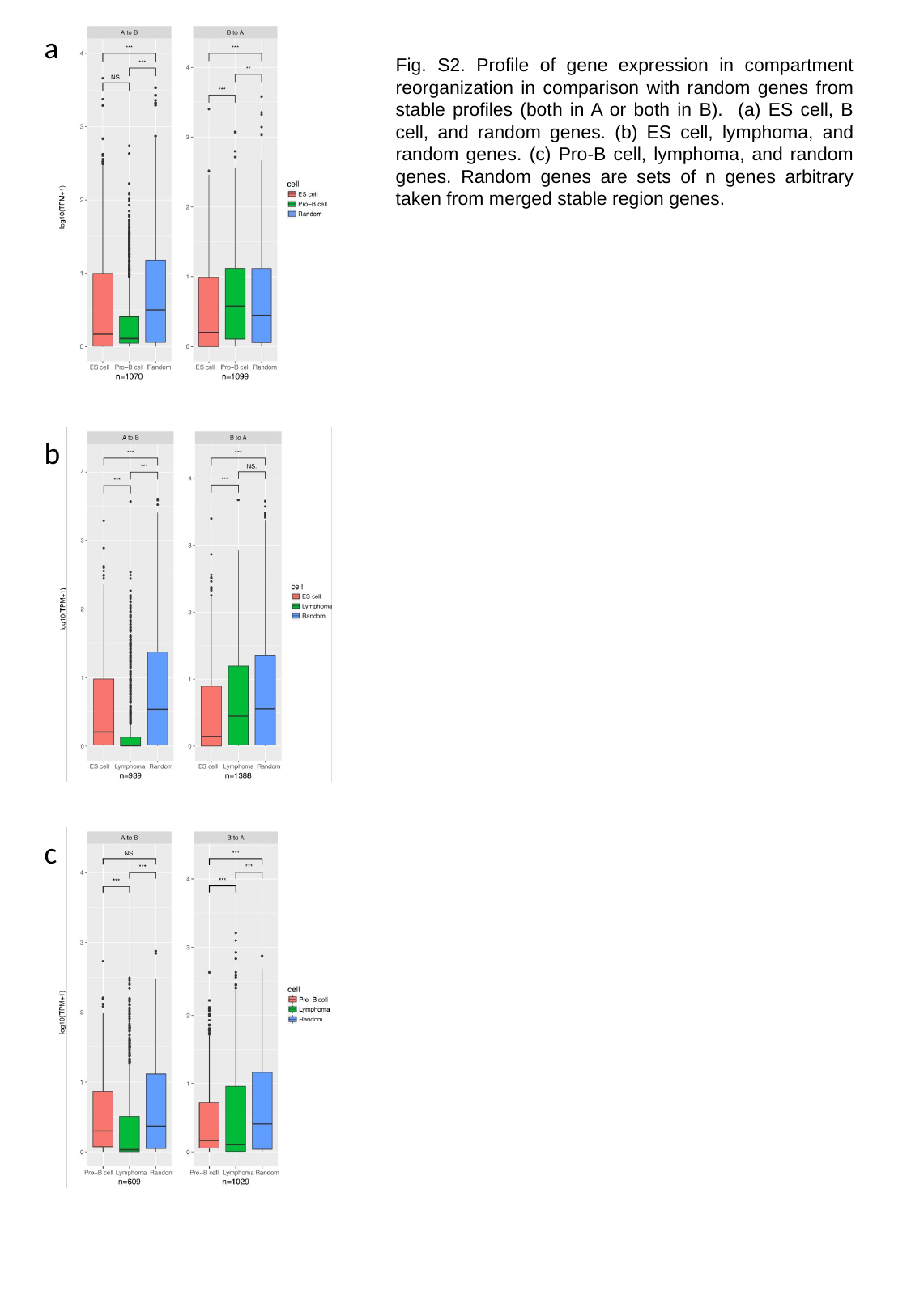

a
Fig. S2. Profile of gene expression in compartment reorganization in comparison with random genes from stable profiles (both in A or both in B). (a) ES cell, B cell, and random genes. (b) ES cell, lymphoma, and random genes. (c) Pro-B cell, lymphoma, and random genes. Random genes are sets of n genes arbitrary taken from merged stable region genes.
b
c

## Slide 3
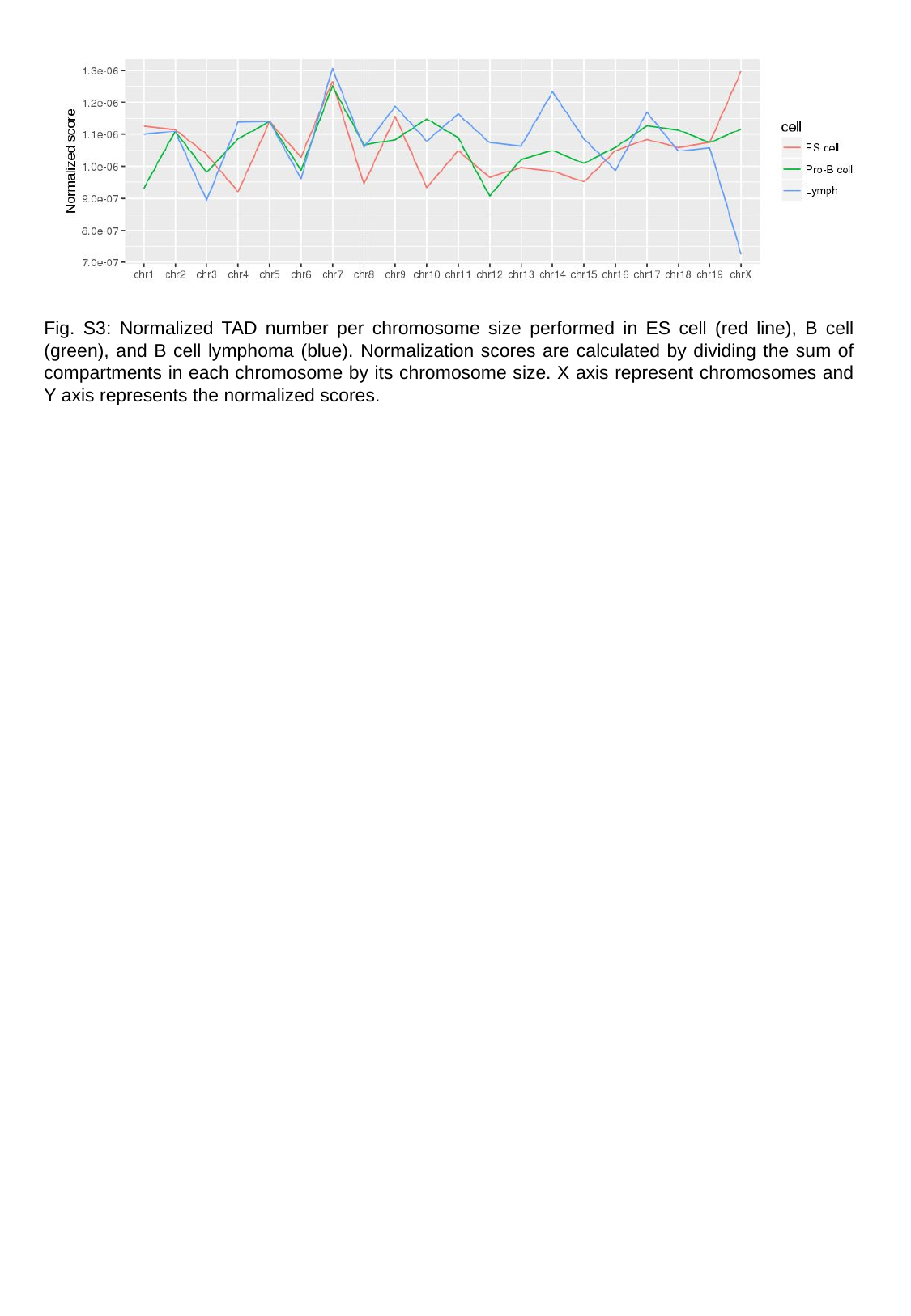

Fig. S3: Normalized TAD number per chromosome size performed in ES cell (red line), B cell (green), and B cell lymphoma (blue). Normalization scores are calculated by dividing the sum of compartments in each chromosome by its chromosome size. X axis represent chromosomes and Y axis represents the normalized scores.

## Slide 4
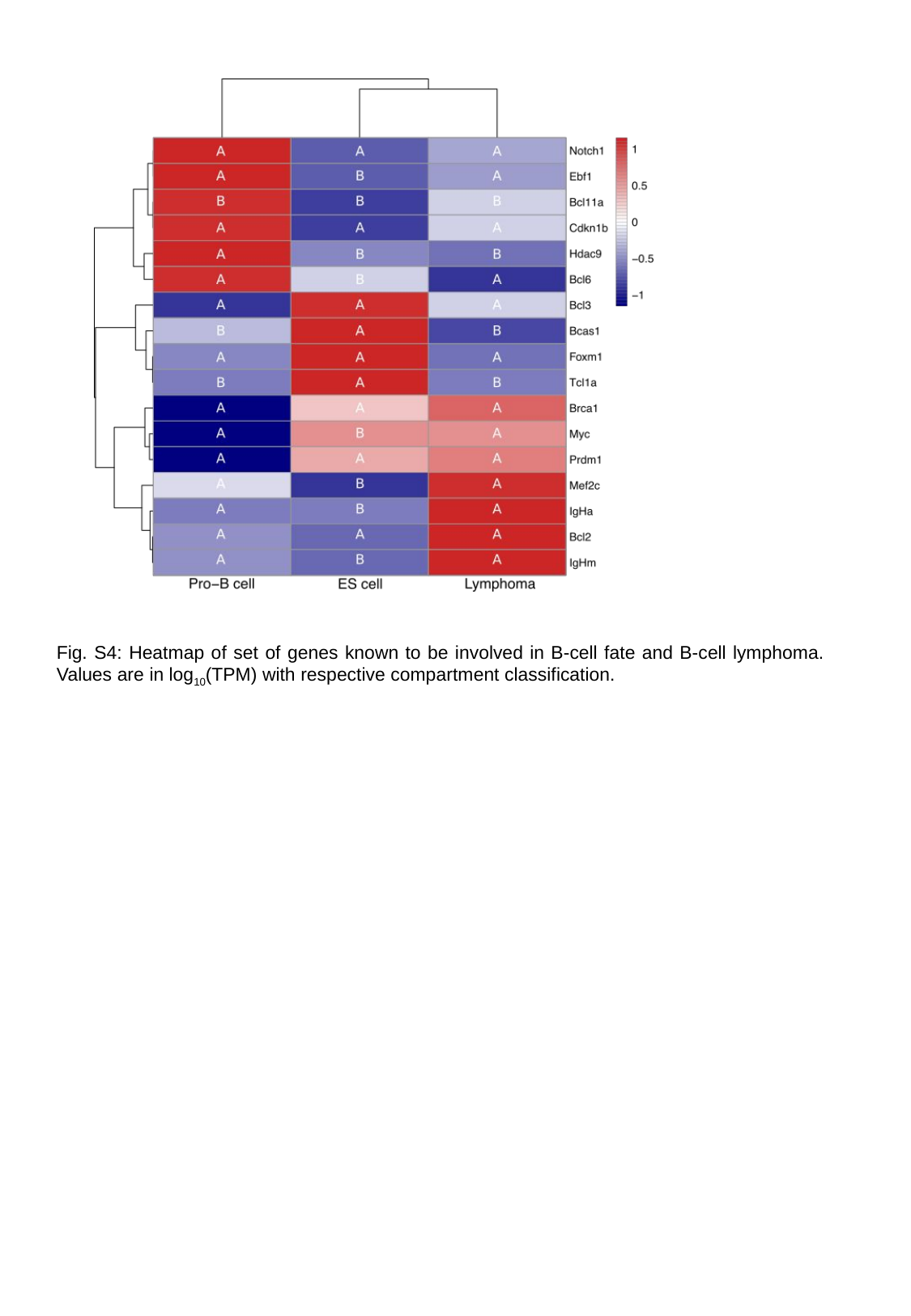

Fig. S4: Heatmap of set of genes known to be involved in B-cell fate and B-cell lymphoma. Values are in log10(TPM) with respective compartment classification.
